# A Systematic Review of Perception System and Simulators for Autonomous Vehicles Research

**DOI:** 10.3390/s19030648

**Published:** 2019-02-05

**Authors:** Francisca Rosique, Pedro J. Navarro, Carlos Fernández, Antonio Padilla

**Affiliations:** División de Sistemas e Ingeniería Electrónica (DSIE), Universidad Politécnica de Cartagena, Campus Muralla del Mar, s/n, 30202 Cartagena, Spain; carlos.fernandez@upct.es (C.F.); antonio.padilla@upct.es (A.P.)

**Keywords:** autonomous vehicle, perception system, simulator, LiDAR, model based design

## Abstract

This paper presents a systematic review of the perception systems and simulators for autonomous vehicles (AV). This work has been divided into three parts. In the first part, perception systems are categorized as environment perception systems and positioning estimation systems. The paper presents the physical fundamentals, principle functioning, and electromagnetic spectrum used to operate the most common sensors used in perception systems (ultrasonic, RADAR, LiDAR, cameras, IMU, GNSS, RTK, etc.). Furthermore, their strengths and weaknesses are shown, and the quantification of their features using spider charts will allow proper selection of different sensors depending on 11 features. In the second part, the main elements to be taken into account in the simulation of a perception system of an AV are presented. For this purpose, the paper describes simulators for model-based development, the main game engines that can be used for simulation, simulators from the robotics field, and lastly simulators used specifically for AV. Finally, the current state of regulations that are being applied in different countries around the world on issues concerning the implementation of autonomous vehicles is presented.

## 1. Introduction

Rapid advances in electronics, information, and communications technology (leading to miniaturization and improvement of computers, sensors and networking performance) have given rise to the development of several autonomous vehicles (AV) technologies [1]. UCSUSA [2] defines autonomous vehicles as follows: “Self-driving vehicles as cars or trucks in which human drivers are never required to take control to safely operate the vehicle. Also known as autonomous or “driverless” cars, they combine sensors and software to control, navigate, and drive the vehicle.” For Thrun [3], an AV is “an unmanned vehicle that is capable of sensing its environment and navigating without human input”. However, the standard way to discuss autonomous vehicles is to talk about “self-driving levels”, as defined by the SAE (Society of Automotive Engineers) [4]. The SAE, which is an automobile standardization agency, divided the autonomous driving capacity of a vehicle into six levels, from the most basic systems to 100% autonomous driving. These levels help measure how advanced the technology of a certain autonomous car is. This has opened up numerous fields of research and development that, although end up being interconnected, correspond to very diverse areas.

In parallel to this evolution, the processes and procedures (function requirements and regulations) established for testing AV functions have also been developed and established over the previous decades. There, however, are concerns regarding the possible consequences of such a technology, especially with regard to peoples’ safety, mechanical failures that may cause crash and the costs of such an incident. To guarantee that an AV is safe and to reduce costs, different scenarios must be modelled and tested. Systematic testing of autonomous vehicles can be performed in simulation or in the physical world. Physical tests offer real testing scenarios, and engineers can use actual vehicles instead of models. However, regulations restrict the use of these vehicles in cities; thus, to perform tests with real AVs, one needs access to expensive hardware and field tests, which consume a considerable amount of time. According to recently published reports [5,6], it is impossible to perform empirical field tests that verify the safety of autonomous cars in a reasonable timeframe. In this context, simulation, modelling and testing has the potential to fill the gap and enable rigorous, controlled, and timely evaluation AV systems. 

The research community recognizes three types of simulations [7]: live, virtual, and constructive (LVC). A live simulation is simply an operational test, with sensors used to identify which systems have been damaged by simulated firings, using real forces and real equipment. It is the closest exercise to real use. A virtual simulation ("X-in-the-loop") might test a complete system prototype with stimuli produced either by a computer or otherwise artificially generated. This sort of exercise is typical of a developmental test. A constructive simulation is a computer-only representation of a system or systems. Therefore, a simulation can vary from operational tests to a fully computer-generated representation (i.e., no system components involved) of how a system will react to multiple inputs. It can be used for several purposes: (1) The design and evaluation of operational and development tests; (2) simulation, taking into account the levels of system aggregation, modeling level of the individual components of a system (for example, the system software), or modeling level of the system as a whole—to model a complete prototype and model multiple system interactions (i.e., a RADAR component with the rest of the system).

**Figure 1:**
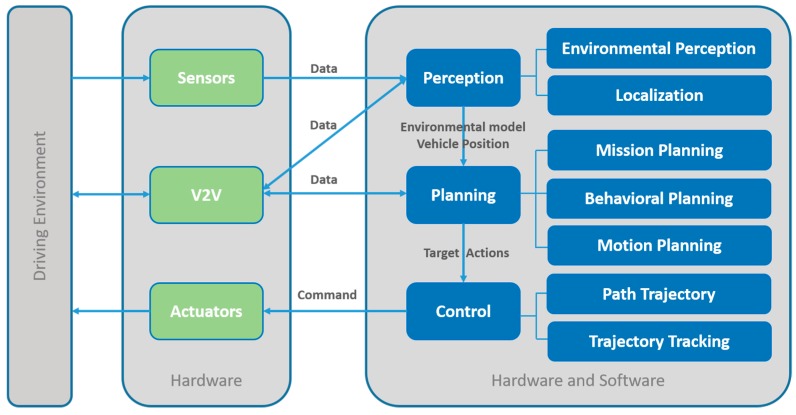
Typical autonomous vehicle system.

However, as is exposed in [8], the first thing that must be taken into account is the type of characteristics or systems that can be tested through simulation (see Figure 1). In this work, we focus on the perception of autonomous vehicles as offering greater autonomy and complexity, emphasizing on their subsystems [9]: Environmental perception, and localization. 

In this paper, we present a systematic literature review on simulators applied to system perception in autonomous vehicles. For this, the technological and legacy aspects involved in the development of AVs are presented in detail, specifying the different alternatives available to simulate and test each of the subsystems.

The paper is organized as follows: Section 2 and Section 3 detail the technologies habitually engaged to capture information from the environment and to estimate position of the autonomous vehicles. Section 4 discusses the current fusion technics used to combined information from the perception systems. Section 5 carries out an exhaustive collection of tools and platforms capable of performing simulation tasks in the AV ambit. Section 6 shows the current state of legislation with regard to the legal aspects of autonomous driving and testing possibilities of AV in different countries. Section 7 presents our conclusions.

## 2. Environment Perception System

An autonomous vehicle acquires knowledge of its surrounding in two stages. The first stage consists of scanning the road ahead to detect possible changes in driving conditions (traffic lights and signs, pedestrian crossing, and barriers, among others). The second stage relates to the perception of other vehicles.

**Figure 2:**
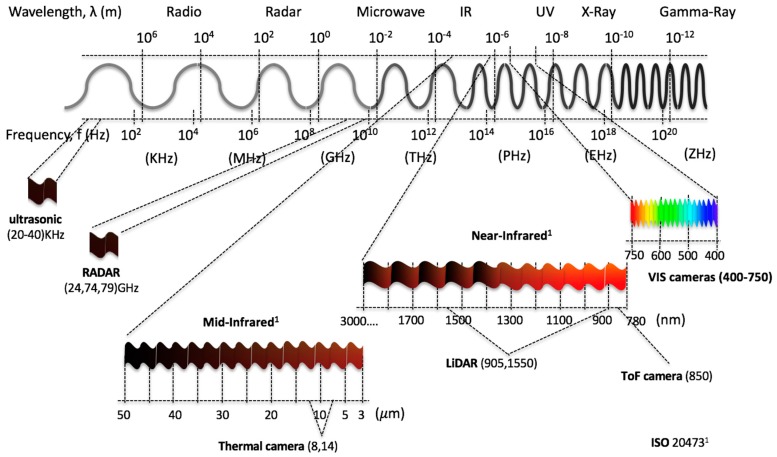
An overview of different spectra used for perception systems in autonomous vehicles.

This section presents the most representative sensors that make up the perception systems of AVs: Ultrasonic, RADAR, LiDAR, cameras, IMU, GNSS, and RTK. There are numerous informative and scientific articles or books that show the types of sensors used in AVs, their applications, as well as their advantages and disadvantages. These works portray the sensors as black boxes, formed by a set of inputs and outputs, without delving into the physical foundations of their operation. As a novel aspect of this work, these sensors are presented from a point of view of the electromagnetic spectrum that they actively or passively use for their operation. This will allow researchers to acquire a deeper knowledge of the benefits and disadvantages of these sensors in degraded environments or adverse weather conditions. In Figure 2, the electromagnetic spectrum is divided into two scales: Wavelengths and frequencies. In addition, the spectral ranges used by the sensors analysed in this work are shown.

### 2.1. Ultrasonic Sensor

As its name indicates, ultrasonic sensors use sonic waves, in the range of 20 kHz to 40 kHz, generated by a magnetoresistive membrane, to measure the distance to an object. Its principle of operation is based on the measurement of the time of flight (ToF) of the sonic wave from when it is emitted until the echo is received:

(1) $$d=\frac{c}{2}\times \mathrm{ToF}$$

The *c* velocity of the wave is in meters per second and ToF is time of flight in seconds.

**Figure 3:**
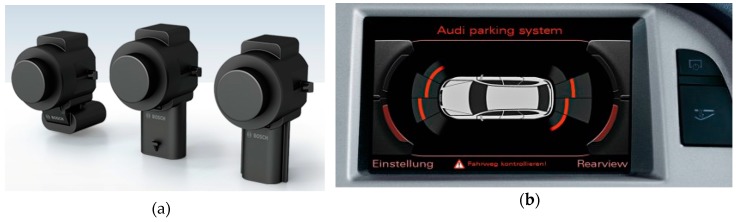
(**a**) Ultrasonic car sensors from Bosch; (**b**) an assistance parking system from Audi.

These sensors are usually used in industrial environments for the measurement of height in storage of all types of raw materials. In vehicles, they are used in parking systems or as short distance measurement sensors at low speeds (Figure 3a,b). These low-cost sensors produce good results when measuring distances with any material, independent of its colour, in dusty environments or in adverse weather conditions (humidity or rain). Disadvantages of these sensors include a tendency to produce false positives by bouncing, and a blind zone (blanking) in the measurements, located between the sender element of the sensor and the minimum range.

### 2.2. RADAR (Radio Detection and Ranging)

**Figure 4:**
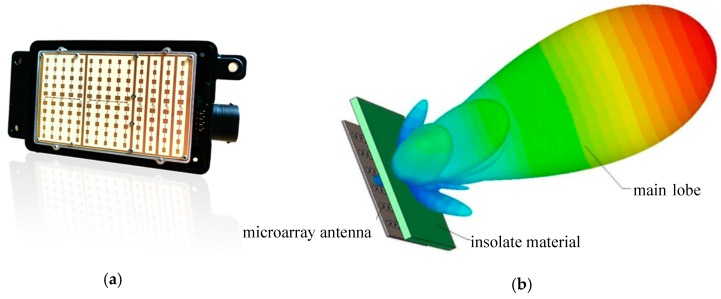
Millimetre-wave RADAR CAR70 from Nanoradar: (**a**) Microarray radar antenna; (**b**) multi-lobe system.

Radar systems work in wavelengths of the order of millimetres; these are used in a wide variety of military and civil applications, such as aerial or terrestrial threat detection systems, shooting systems, and airports or meteorological systems. The emergence of smart vehicles and the need to increase road safety have triggered the use of this type of device in the automotive sector. Radar systems for intelligent vehicles work at frequencies of 24/77/79 GHz, known as millimetre wave radar (MMW). The radar measures the distance between the emitter and the object by calculating the time of flight of the emitted signal and the received echo. The radars not only allow the detection of the distance to several targets, but are also capable of accurately supplying the direction and speed of the targets. The new radars for vehicles use an array of micro antennas capable of generating a set of lobes that allow improvement of the range and a processing system for the detection of multiple targets (Figure 4a,b). 

Range millimetre-wave RADAR is applied in Blind Spot Detection (BSD), Lane Change Assistant (LCA), Rear Cross Traffic Alert (RCTA), Forward Cross Traffic Alert (FCTA) or radar video fusion. Radar waves have higher penetrability because they offer good features in all weather conditions, and can accurately detect short-range targets in front, to the side, and to the rear side of vehicle. For this reason, they are used in several ADAS systems. RADAR can significantly improve vehicle safety performance and reduce the decision-making burden of people at the wheel. Furthermore, it can be installed besides the bumpers of the vehicle. Some disadvantages of this sensor type are the lack of precision, its reduced Field of View (FOV), and the fact that it can produce false positives due to bouncing of the emitted signal.

### 2.3. LiDAR (Light Detection and Ranging)

**Figure 5:**
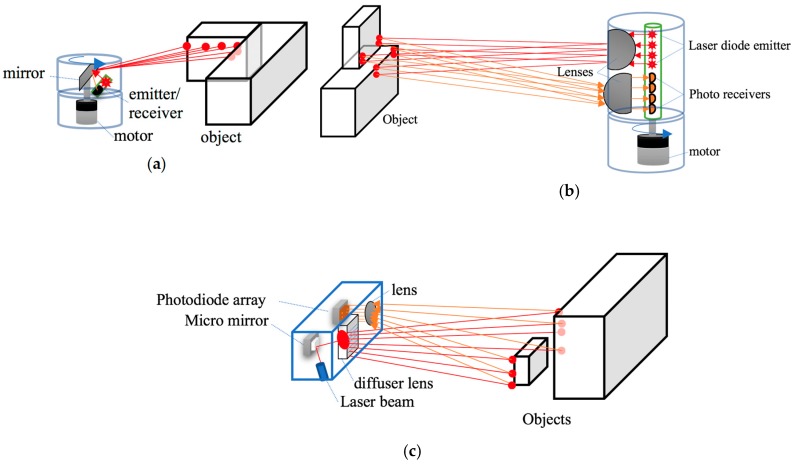
Operating schemes: (**a**) Rotating 2D LiDAR, (**b**) rotating 3D LiDAR, (**c**) solid state 3D LiDAR.

LiDAR systems were initially developed in the 70s to measure elements in sea or land from satellites or airplanes. It was specifically developed for the detection of submarines by the American Navy. LiDAR systems base their operation on the measurement of the time of flight of a pulsed light emitted from a laser diode until it is received by an emitter. The emission are in infrared ranges (905 nm or 1550 nm). Emissions at 905 nm require less energy than those emitted at 1550 nm because the water in the atmosphere begins to absorb energy from 1400 nm. This initial disadvantage of power increase at 1550 nm is used by the aqueous liquid of the eye to totally filter this wavelength, making them less harmful than LiDAR at 905 nm [10]. Lasers used for vehicles belong to Class 1 [11] and are safe under all conditions of normal use. LiDARs use the ToF principle to carry out the measurement of distance between emission and reception. These can be classified according to the type of information they obtain from their environment in 2D or 3D LiDARs or it can be classified according to their construction rotary or solid state LiDAR. 2D LiDAR obtains information from the environment by projecting a single laser beam on a rotating mirror perpendicular to the axis of rotation (see Figure 5a). The 3D LiDAR allows to obtain a 3D map of great accuracy to be obtained of the environment; for that purpose, they use a set of diodes lasers mounted on a pod that rotates at high speed (see Figure 5b). The number of lasers installed in the pod determines the accuracy of the point cloud obtained in each turn. Currently we can find 3D LiDARs that integrate from 4 to 128 lasers or channels with a horizontal FOV of 360 grades and vertical FOV that oscillates between 20–45 grades with accuracy of a few centimetres. Depending of the number of channels, 3D LiDAR are used in Adaptive Cruise Control (ACC), object avoidance, object identification or 3D mapping. LiDAR is affected by weather conditions such as rain, snow, fog or dusty environments due to the diffraction of light in these environments. Furthermore, they reduce their operating range detection depending on the reflectivity of the objects that are reached by the laser beams. The maximum detection capacity depending on the type of reflectivity of the material to be detected is presented in the datasheet provided by the manufacturer.

The last kind of device based on laser measurement that has arrived in the autonomous vehicles world is the solid state LiDAR. Solid state LiDAR allows a 3D representation of the scene to be obtained around the LiDAR without the use of mobile parts in the device. A micro mirror MEMS circuit carries out the synchronization with a beam laser to scan the horizontal FOV in multiples lines. For that, the micro-mirror reflects the beam over a diffuser lens, which creates a vertical line that touches the objects (Figure 5c). The light reflected is captured by a lens and is sent to a photodetector array to build the first line of a 3D matrix. The process is repeated until a point cloud of the scene is created. This feature notably increases its durability, reduces maintenance tasks, and decreases its price. Solid state has a smaller FOV than the rotary LiDAR. The trends in perception system are replacing the current rotating 3D LiDAR by a set of solid state LiDARs integrated around the vehicle. 

### 2.4. Cameras 

In the perception system of autonomous vehicles and from a point of view of the wavelength received by the device, cameras can be classified as visible (VIS) or infrared (IR). The element used by the camera to capture a scene is known as an imaging sensor and has traditionally been implemented with two technologies: Charge-coupled device (CCD) and complementary metal oxide semiconductor (CMOS). CCD image sensors are manufactured by an expensive manufacturing process that confers them unique properties such as high quantification efficiency and low noise. CMOS was developed to reduce the cost of manufacturing at the expense of reducing its performance. The design of the extraction architecture of the luminosity values allows the selection and processing of regions of interest (ROI); furthermore, the CMOS device has a lower consumption than CCDs. These characteristics make them the most used technology for mobile devices. On the other hand, CCD technology has a high dynamic range and higher image quality in low light environments. The differences of both technologies begin to overlap and it is expected that in the future, CMOS technology will replace CCD [12,13].

VIS cameras capture wavelengths between 400 nm to 780 nm (see Figure 2), same as the human eye can. The visible spectrum is divided into three bands or channels: R, G and B, which will be coded separately. These devices are the most commonly used in AV perception systems to obtain information about the surroundings of the vehicle due to their low cost, high quality colour information, and high resolution. The huge volume of data generated by means of the device supposes a further problem for the processing system. The most common applications are BSD, side view control, accident recording, object identification, LCA, and signs detection. VIS cameras are highly affected by variations in lighting conditions, rain, snow or fog conditions and for this reason are combined with RADAR and LiDAR technologies to increase its robustness. 

The combinations of two VIS cameras with a known focal distance allows stereoscopy vision to be performed, which adds a new channel called depth information. Cameras with these features are known as RGBD. These devices supply a 3D representation of the scene around the vehicle.

IR cameras are passive sensors that work in infrared (IR) wavelengths ranges between 780 nm to 1 mm. There are many devices which work in this spectrum because fewer light interferences exist (e.g., LiDARs). Perception systems that includes IR cameras [14,15] work in near-infrared (NIR: 780 nm–3 mm) or mid-infrared (MIR: 3 mm–50 mm, known as thermal cameras) ranges. The uses of NIR usually replace or complement VIS cameras. IR cameras are used: (1) In situations where there are peaks of illumination; for example, at the exit of a tunnel, when driving in front of the sun or when long light crosses the car; and (2) in hot body detection, such as pedestrians [16,17,18], animals [19] or other vehicles [20]. In these cases, the thermal cameras allow the segmentation process to be simplified to fewer operations based on thresholds and they are not affected by weather or lighting conditions. On the other hand, they supply a grey scale image and the bigger cell size of the image sensor notably reduces its resolution.

**Figure 6:**
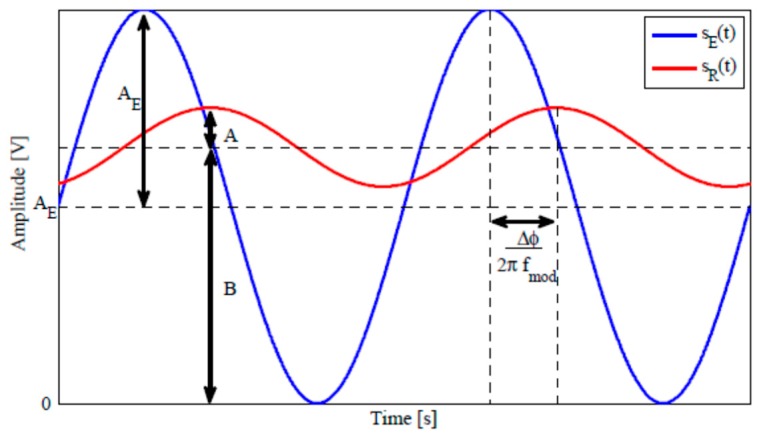
Emitted signal (blue) and received signal (red).

ToF cameras are active sensors that use the time of flight principle to obtain a 3D representation of the objects in the scene. ToF cameras emit NIR light pulses of 850 nm with an LED (Light Emitting Diodes) array and they measure the difference in phase Δφ between the modulated signal emitted (s_E_) and the signal received (s_R_) to compute the distance, as shown in Equation 1 and Figure 6 [21].

(2) $$d=\frac{c}{2}\times \frac{\Delta \varphi }{2\pi f_{mod}}$$

The distance ranges from 10 m for indoor scenes and about 4 m for outdoor scenes, depending on the number of LEDs in the matrix. As with IR cameras, they have a low resolution due to characteristics of the wavelength they are required to capture.

Table 1 shows a summary of the advantages and disadvantages of the sensors analysed in this section. In the table are shown 11 features, which have been quantified in line with data obtained in this review. The quantification has been carried out using four scores to simplify the process: 0—none, 1—low, 2—medium and 3—high. The first six features pose maximum quality with the highest score and the rest obtained the best quality with the minimum score.

**Figure 7:**
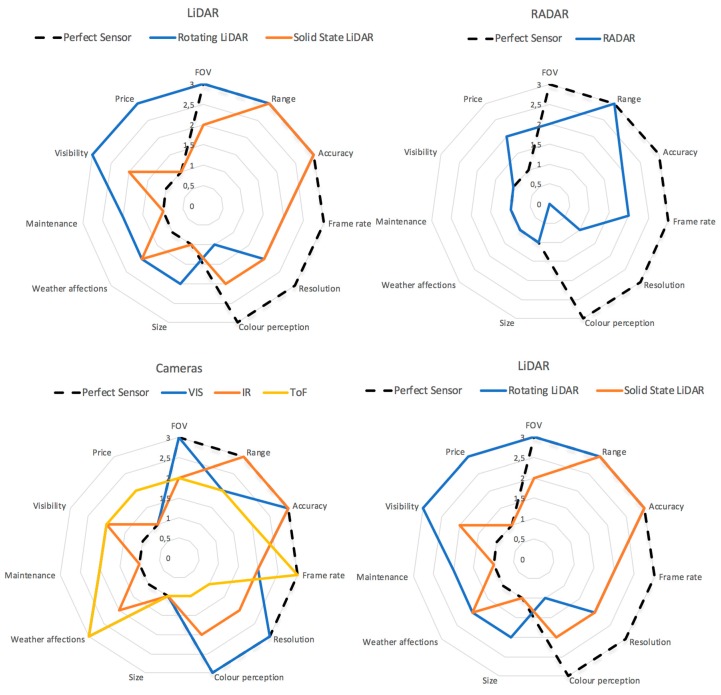
Comparison of the features of the different sensors used in environment perception systems.

Figure 7 shows a set of spider charts of features of the sensors presented in this review. A perfect sensor is defined as the one that obtains the best scores in all the characteristics analysed in this review. This means maximum values (3) for FOV, range, accuracy, frame-rate, resolution, colour perception, and minimum values (0) for weather affections, maintenance, visibility, and price. This comparison offers a clear overview of the sensors’ strengths and weaknesses.

## 3. Position Estimation Systems

### 3.1. Global Positioning Systems 

Global Navigation Satellite System (GNSS) is the most widely used technology for vehicle positioning on land, sea, and air. The GNSSs provides the absolute position of a receiver with respect to a fixed reference and consists of a set of satellites orbiting approximately 20,000 km from the earth’s surface. These emit signals with information about the satellite, its position, orbital parameters, etc. This system is complete with reception systems, which receive those signals and extract information about position, speed, and exact time. The best-known GNSS system is the Global Positioning System or GPS, developed by the USA in the 1970s, which consists of 24 satellites located in six planes separated by 55º and with a rotation period of 11 h and 58 min. Its configuration allows any receiver located on the earth’s surface to receive signals from between 6 and 12 satellites.

The operating principal of the GNSS is based on measuring the time of flight of the signal emitted by the satellite and that received by the receptor. The system is able to reliably obtain details of position and time (x, y, z, t) with a minimum of four visible satellites. Table 2 shows the characteristics of the most widely used GNSS systems for global positioning, their constellations, precision, coverage, period of rotation, height, and owner [22,23].

Satellite signals are influenced by numerous errors: (1) Synchronization of the atomic clocks of the orbiting satellites, (2) signal transmission by the ionosphere and troposphere, (3) noise in the receivers, (4) multipath effect due to signal reflections, and (5) geometric uncertainties.

Differential GNSS (DGNSS) was developed to alleviate errors that affect signal measurement from satellite constellations. The DGNSS consists of two GNSS receivers, a ground or base station and a mobile station, known as a Rover. The base station knows its exact position and continuously communicates the signal corrections to the moving station. The signal corrections provide precisions of 0.7 m to 3 m in civil applications and improve the integrity of the measurement (ability of the system to provide timely warnings to users when the system should not be used for navigation). DGNSS needs a system of precisely georeferenced land stations and a communication system, usually UHF radio, with the mobile stations. 

There are other systems based on signal correction that come from satellites, such as the Satellite Based Augmentation System (SBAS) and Real-Time Kinematic (RTK).

- SBAS was designed to improve air navigation by increasing the horizontal and vertical accuracy of the receptors and providing information about the quality of the signals. For this purpose, it has a set of stations distributed over large geographical areas that monitor the status of the satellite constellations, informing of any anomaly. These systems are operated by different institutions, governments, and even private companies [24].
- RTK systems use the satellite signal carrier to improve the position accuracy of the mobile stations; the base station retransmits the carrier to one or more rover stations and these compare the carriers with the signals received from the satellites and calculate the position accurately. These systems obtain precisions of up to 2 cm and usually use a radio modem to communicate between base stations and rovers within a range of 20 km. RTK has been successfully applied in autonomous driving [25] and precision agriculture [26].

### 3.2. Dead-Reckoning (DR) and Inertial Positioning 

DR is the process of estimating the position and heading of a vehicle based on previous position measurements. The simplest position estimates are made using rotary sensors (encoders) fixed to the steering wheel and the wheels of the vehicle [27], a technique known as odometry. It is not capable of quantifying slippages or lateral movements of the vehicle, and for this reason is complemented with Inertial Measurement Units (IMUs) which combine accelerometers, gyroscopes and magnetometers [28]. The incorporation of these sensors allows the previously mentioned errors to be corrected and the sampling speed of the measurement system to be increased. However, they introduce errors due to the measurement of first and second order variables [29]. IMUs alone do not provide a global vehicle position, so they are usually accompanied by a GNSS system. 

The current positioning systems for vehicles are hybrid systems that merge data from different sources such as odometry, IMUs, GNSS, LiDARs, RADARs and cameras to obtain a reliable position and heading with tolerable error.

## 4. Fusion Algorithms 

Sensory fusion or data fusion aims to improve the measurement of two or more sources of data from sensors, beyond the individual measurement of each of them. Sensory fusion is especially indicated when large amounts of disparate sensor data are produced. Sensorial fusion applied to the measurement of redundant data reduces the uncertainty of the measurement, increases the accuracy and improves the integrity of the system, improving fault tolerance. 

Obtaining a classification of the algorithms or fusion techniques is an arduous and difficult task due to multidisplinarity and the large number of case studies reported in the literature. In a review carried out by [30], it is possible to find an extensive classification of fusion methods according to different criteria such as: (1) relations between the input data sources; (2) input/output data types and their nature; (3) abstraction level of the employed data; (4) different data fusion levels, and (5) architecture types. In this review, we will stablish three categories: (1) estimation methods based on Gaussian filters (e.g., Kalman filter (KF) or particle filters (PF)), (2) probabilistic inference methods (i.e., Bayes theorem), and (3) artificial intelligence methods based on machine learning algorithms. Three categorizations are applied to both perception systems and location systems in autonomous vehicles. Following are examples that combine the merging of data from the different categorizations. and different sensors of the perception and location systems.

### 4.1. Fusion Methods in Perception Systems

There are numerous works related to the sensors mentioned in this review that detect vehicles, pedestrians, lanes, signs, and so on. In [31], the data supplied by a LiDAR and a stereo camera are fused to improve a vehicle’s detection. The method has two stages: (1) Hypothesis generation, where a vehicle candidate is obtained, combining Haar features from depth maps with the AdaBoost classifier; and (2) hypothesis verification, where a shape estimation of the candidates is calculated using the LiDAR information and a support vector machine. The proposed fusion approach achieves a lower false alarm rate in urban environments. A method that fuses the MMW RADAR with camera information is presented in [32]. The work presents a collaborative fusion approach to achieve an optimal balance between vehicle detection accuracy and computational efficiency. The MMW radar first detects the potential vehicle and provides a region of interest. The vision processing module employs symmetry detection and active contour detection to identify the vehicle inside the region of interest provided by the MMW radar. The experimental results show a 92.36% detection rate and 0% false alarm rate using a real-world dataset. In [33], the advantages of the fusion of LiDAR and RADAR sensors are used in order to provide permanent precise spatial and dynamical data. The work presents a real-time algorithm, which enables an autonomous car to comfortably follow other cars at various speeds, while maintaining a safe distance. In [34], an unscented Kalman filter and Joint Probabilistic Data Association is used to fuse the data from a 2D LiDAR and camera information to detect vehicles. The presented approach achieves an increase in safety for vehicle detection in single-lane carriage-ways, where casualties are higher than for other road classes.

The increase in precision and robustness obtained with the fusion methods when redundant information is available is especially relevant when the systems can produce injuries or death to people. Pedestrian detection is a common area to use the fusion algorithms, as demonstrated by numerous works found on this topic. In [35], the distance data and the reflexivity of a 3D LiDAR are fused through a set of 50 features to detect pedestrians. The features are composed by three subsets: (1) Shape features obtained in XYZ projections of pedestrian cloud points, (2) Hu invariants moments from XYZ projections and, (3) statistical features from the reflexivity data. The data fused was used to train an SVM. The pedestrian detection method obtained a higher classification rate when it was compared with similar algorithms. In [36], a set of machine-learning algorithms (MLA) are tested with different fusion schemes with the proposal of determining the best performance in pedestrian detection. The experimental results provide a false positive rate, AUC (Area Under Curve ROC is a metric to evaluate the performance of two classifiers), and provide an accuracy of 96.67%. An RGB camera and a LiDAR are fused for pedestrian detection. LiDAR is used to evaluate the value of depth perception for pedestrian detection. In the work, the detectors were trained on both input modalities (from KITTI database) and various fusion strategies. The best performance was obtained by a late re-scoring strategy that was designed to be sensitive to geometric context. The advance in GPUs has allowed algorithm execution times that would have been unthinkable a few years ago (i.e., CNN); there are numerous projects that use convolutional neural networks (CNN) to obtain high grades of data abstraction. In [37], multispectral information is used from a thermal camera and an RGB camera (TRGB channels) and a multi-layer fused CNN to detect pedestrians under adverse illumination conditions. The algorithm development on the basis of a multiple-layer fusion technique can significantly reduce the detection miss rate. In [38], the distance data with reflectance information from 3D LiDAR is fused by means of a CNN. Distance and intensity raw data from LiDAR are transformed to high-resolution (dense) maps, which allow direct implementation on CNNs, both as single or multi-channel inputs. The results of the CNN were tested with the KITTI dataset Vision Benchmark Suite [39].

### 4.2. Fusion Methods in Positioning Systems

The precise determination of the position of a vehicle is a crucial aspect for navigation tasks in related areas such as autonomous vehicles, intelligent transporting systems and intelligent vehicles. The bibliography reports a huge number of applications where fusion methods are used with sensors belonging to both positioning systems and environment perception systems mentioned in this work.

Traditionally, the Kalman filter and extended Kalman filter has been one of the most used algorithms to reduce the degree of uncertainty in sets of data supplied by different sources and to increase the accuracy in positioning systems. In [40], a real-time data fusion system for improving car positioning precision in urban environments is proposed. The proposed method uses the data from four-wheel speed sensors and low-cost GNSS with an EKF to fuse the data. The urban scenarios are prone to suffer low precision and transient unavailability of the GNSS signal. The main contribution of this work enables accurate car positioning during short GNSS signal outages. In [41], a fusion framework to obtain the cooperative positioning information from radar sensor data is presented. The framework fuses the information received from radar sensors by means of the KF method. The measurements recorded on a highway and a rural road demonstrate that the fusion of both information sources outperforms the positioning estimation using only the radar sensor 

In [42], a fusion method is presented, which integrates the information supplied by a stereo camera, a 2D LiDAR, and a GPS to obtain precise positioning of a vehicle. The proposed method uses a prelaminar stage, where an outlier-rejection invariant closest point method reduces the matching ambiguities of scan alignment during tasks of motion estimation with the 2D LiDAR. Finally, after the validation process, the information is fused by means of an unscented information filter.

The particle filter (PF) is a method used to obtain a good estimation of vehicle position in non-linear models. In [43], a robust fusion algorithm is presented, based on a particle filter using the entropy information theory. The fusion method uses a 3D urban area mapped previously (point cloud map) together with a 3D LiDAR with 32 channels, an IMU, and wheel odometry for obtaining the location of a vehicle in urban scenarios. The results have been compared with the offline ground-truth obtained with a RTK-GPS. Another example is the location of a vehicle in urban areas by fusing the data and a PF as shown in [44]. In this case, the data input to the particle filter is composed of GPS, an IMU, a camera, and a digital map. The experimental results have shown that this system reliably localizes the vehicle, even while passing through tunnels, long urban canyons, and under an elevated railroad.

The selection of the most suitable type of filter for data fusion will depend on the linearity of the model used. For linear models, the KF provides an optimal solution to the fusion problem. For non-linear models, other techniques such as EKF, UKF, or PF are used to perform the linearization of the model. For example, in the field of perception systems, the non-linearity of the data produced by RADAR systems requires the use of EKF, UKF or PF filters. The bibliography reports comparisons in the performance of this type of filters; the best results in the estimation of non-linear models are produced with UKF [45,46].

Fusion techniques based on features extraction that use classification algorithms (e.g., AdaBoost, SVM, etc.) are being overcome by the enormous degree of data abstraction achieved by CNNs. The ability to model any system, however complex it may be, through the insertion of thousands or millions of hidden layers with different kernels, coupled with the computational increase of the new GPUs, CNN will have a promising future as a data fusion technique.

## 5. Simulation

Modelling and simulation are well-established tools for analysis, design, acquisition and training in the automotive domain. Despite the heterogeneity of subsystems and disciplines involved in the development of an autonomous vehicle [9], there are many simulation methods that together allow covering of the entire development process. V-model and its variants have become the most common process models adopted in the automotive industry, guiding the development of systems on a variety of refinement levels with a multiple-stage validation and testing process [47]. By applying virtual simulation technologies at different abstraction levels, several “X”-in-the-Loop (XIL) (where “X” refers to any type of test included in the development process) testing setups can be performed: Model-In-the-Loop (MIL), Software-In-the-Loop (SIL), and Hardware-in-the-Loop (HIL). Moreover, ISO 26262 itself does have some limited guidance on the use of simulation in verification activities. The safety process of ISO 26262 is based on a safety V-model. As such, it is not straightforward to match with an agile development process, which is the natural choice for AV development. Section 6, “Product Development at the Software level”, provides some directions on verification of the software architecture design and recommends “simulation of dynamic parts of the design”. Section 6 further notes that “software integration testing can be executed in different environments”, and lists as examples MIL tests, SIL tests, processor-in-the-loop tests, and HIL tests.

Although the number of tools for the simulation of autonomous vehicles has increased in recent years, it is very difficult to select the best tool for a specific development. Features like open-source, multi-platform, personalization and documentation are desirable in all simulators. Furthermore, regarding the simulation of such robotic systems, one must take into account all the aspects of physical implementation to further simplify the transition from virtual- to real-world scenarios. 

The Agent paradigm has been introduced as well, as a way to address some of the current issues in autonomous-based driver behavior regarding its distribution capabilities, computing efficiency and scalability [48,49]. However, these are not found in all the simulation tools. Some of the solutions provide a series of toolchains to address the end-to-end process of an autonomous vehicle; others only contemplate some of the subsystems or functionalities (Advanced Driver Assistance Systems (ADAS), Simultaneous Localization And Mapping (SLAM), Driving, traffic, etc.). Most simulation tools are compatible with programming languages such as C/C++, Perl, Python, Java, LabVIEW, URBI or MATLAB.

Below we present the different approaches that can be taken into account to select a simulator for autonomous vehicles (vehicle tests, robotics, game engine, specific development), focusing on the perception subsystem.

### 5.1. Vehicle Test Simulation 

As previously mentioned, achieving high fidelity autonomous driving requires testing of autonomous characteristics in every possible scenario. The design, implementation, and testing of vehicles in a wide range of use cases and in realistic traffic conditions are costly, time-consuming, complicated and, often, not reproducible. The integration of tests with the physical platform in these cases are unnecessarily complex and often carried out in the last stage of the development process. This makes prototype design, implementation, intensive testing and simulation with a real vehicle in the simulation circuit the most effective way to verify and validate the design idea. The simulation of Software-In-the-Loop (SIL) in the laboratory environment offers a safe way to prototype and implement the control and algorithms of vehicles. The incorporation of a vehicle and real sensors in the simulation loop (called Hardware-In-the-Loop or HIL) validates the design and reduces the time required for system verification. 

Test engineering roles are plentiful, and that is because it is not easy to make sure everything works all the time in autonomous vehicles. But there are tools that accelerate the process. These software tools provide environments, templates and architectures to validate anything in the vehicle, be it in a consulting engineering or high-volume manufacturing environment. As has been commented previously, in the engineering of vehicles and therefore in autonomous vehicles, the development based on models is more widely accepted and more concretely the development following model V. This model is composed of different phases of development and testing:

- The first step is a Model-In-the-Loop (MIL) [50] approach, which allows quick algorithmic development without involving dedicated hardware. Usually, this level of development involves high-level abstraction software frameworks running on general-purpose computers.
- The second step is a Software-In-the-Loop (SIL) [51] validation, where the actual implementation of the developed model will be evaluated on general-purpose hardware. This step requires a complete software implementation very close to the final one. SIL testing is used to describe a test methodology, where executable code such as algorithms (or even an entire controller strategy), usually written for a particular mechatronic system, is tested within a modelling environment that can help prove or test the software. SIL testing and simulation can thus be a useful technique for software proving at earlier stages of the design.
- The last step of this validation process is Hardware-In-the-Loop (HIL) [52], which involves the final hardware, running the final software with input and output connected to a simulator. HIL testing provides a way of simulating sensors, actuators and mechanical components in a way that connects all the I/O of the Electronic Control Units (ECU) being tested, long before the final system is integrated. It does this by using representative real-time responses, electrical stimuli, and functional use cases. Therefore, the integration of SIL and HIL in the simulator will allow designers and engineers to evaluate advances in the development cycle of the vehicle before the physical prototypes are built.
- Another interesting solution that combines nearly all the advantages of the previous methods without most of their drawbacks is the Vehicle-Hardware-In-the-Loop (VeHIL) approach. This kind of test is a combination of the HIL and test-drive approaches. Functional as well as integration tests can be done easily and early in the development cycle. As the vehicle is physically locked on the chassis-dynamometer, this system greatly improves the safety of the tests.

Although this type of V model development is applied to the entire development process of an autonomous vehicle, it is mainly used in the development of Advanced Driver Assistance Systems (ADAS), where more references to it are found. [47,53,54,55,56]. Most of the HIL tests of the ADAS functions are reduced to sending a series of simulated objects to the unit under test. Depending on the sensor systems involved, information on the kinematics of the object can be included, as well as whether the object is a vehicle, a pedestrian, or something else. In addition to the current sensor data, supplementary vehicle data from other ECUs may be required. Depending on the configuration, several real ECUs can be part of the test bed and connect through automotive network systems such as CAN, FlexRay or Automotive Ethernet. A consortium called ADAS iiT [57] demonstrated a recent example of a HIL test system that uses the platform-based approach for sensor fusion testing. This group demonstrated an ADAS test configuration that can synchronously simulate RADAR, LiDAR, communications and camera signals for an ADAS system. In one case, the configuration was able to simulate a virtual test unit using the CarMaker software (by IPG Automative) and VeriStand (by National Instruments).

To select an XIL simulator, several factors must be taken into account:

- Availability and compatibility of models: The main companies provide you with convenient and powerful solutions to run complex physical models designed with Simulink [58], Stateflow [59], Simscape [60], or any other MathWorks [61] software tool on highest performance multi-core CPUs, GPU and FPGAs. The majority of companies offer Open Models; on other occasions, it will be necessary to develop a custom model or buy one.
- Subsystems which can be tested: simulators were built according to a specific purpose. For example, a Micro HIL system offers a simpler and more economical solution; the strategy is restricted to the analysis of ECU outputs, when excited by specific controlled inputs.
- Real-time simulation communications protocols available, including CAN, FlexRay, ARINC 429, MIL-STD-1553, EtherCAT, real-time UDP and XCP.
- Compliant with ISO 26262: The development of high-integrity systems within the automotive industry is characterized by demonstrating compliance with ISO 26262, an international standard for road vehicle functional safety.

To perform this type of simulation, many of the works found in the literature mainly make use of MathWorks tools [62]. MathWorks is the leading developer of mathematical computing software. MATLAB, the language of technical computing, is a programming environment for algorithm development, data analysis, visualization, and numeric computation. Simulink is a graphical environment for simulation and Model-Based Design for multi-domain dynamic and embedded systems. In particular, Simulink models are used in the development of most pre-ADAS vehicle controllers, and can even be deployed directly to ECUs (following a code generation process). Recently Matlab extended its capabilities for ADAS development with the Autonomous Driving Toolbox, available from the 2017b release. This toolbox provides algorithms and tools for designing and testing ADAS and autonomous driving systems. It allows engineers to automate ground-truth labelling, generate synthetic sensor data for driving scenarios, perform multi-sensor fusion, and design and simulate vision systems. 

Table 3 shows some of the simulators or simulation platforms used for the validation and testing of autonomous vehicles, following a model-based approach.

### 5.2. Games and Physic Engines for Simulation 

A common alternative to using a dedicated simulator for vehicle and robot simulation is to repurpose an available game engine for simulation and research [67]. However, in general, any available game engine could conceivably be used for the purpose of simulation.

A game engine is the software part of a computer game that contains a 2D or 3D graphic representation (rendering engine), representations of physical laws (physics engine), or collision detection (and collision response), sound, scripting, animation, artificial intelligence, networking, streaming, memory management, threading, localization support, scene graph, and may include video support for cinematic. The most modern game engines also include support for Virtual Reality (VR) simulation. Game engines are generally independent of the specific scenarios or applications for which they were originally developed, and the source code of some game engines is partially open. Therefore, researchers can use the source codes of the game engine to create completely new scenarios and applications.

Some of the key features that game engines provide, which are favorable to robotics and autonomous vehicles researchers [68], are listed below:

- Physical fidelity: Realistic simulation, suitable for virtual-reality environments, such as a driving simulator; most recent game engines feature both rigid and soft body dynamics, some of them even use a new dedicated hardware named Physics Processing Unit (PPU). Cutting-edge lightning effects, polygon rendering, and realistic destructible environments are also present/considered
- Distributed architecture: Support for multiple processor cores is included in earlier frameworks for maximum computational resources exploitation. It is possible to simulate multiple entities in multiple networked computers, distributing the processing power over all nodes.
- Cutting-edge graphics: Use of game engines will significantly increase the level of detail and realism of the environment; relating to camera sensor simulation, higher resemblance from the virtual to the real world can be achieved.
- Scriptable environment: Featuring simple but powerful scripting languages, game engines can be rapidly extended to support a new type of sensor, or an optimized statistics module

The main problem with game engine simulators is that they may not be high fidelity. A game engine allows you to test the dynamics based on behavior, but when a high-fidelity simulation is required, you must use models or software that contains a mathematical representation of the subsystems to achieve realistic calculations. This software is often validated with HIL tests, used to a large extent in the evaluation of computer-based test equipment. The high-level algorithms for trajectory planning, vision processing and interactions of multi-agent systems are examples of suitable fields for use with simulators based on game engines.

The principal game engines used in the development of autonomous vehicle simulators, or for some of its subsystems, are: 

Unity 3D [69] is an open source Game Engine, which is primarily used to develop video games and simulations for computers, consoles and mobile devices. The Unity graphics engines use OpenGL, Direct3D, OpenGL for Embedded Systems (OpenGL ES) for mobile platform (iOS, Android) and various APIs. The Unity engine provides built-in support for PhysX physics engine with real-time cloth simulation on skinned meshes, collision layers, and thick ray casts.

Unreal Engine [70], is, like Unity, a popular general-purpose games development engine. It provides a scripting engine, physics engine, and highly realistic video capabilities

Blender [71] is an open source 3D modelling and rendering application whose main purpose is the creation of computer generated images and animations. Though it is not designed as a tool for simulation, it provides many features that facilitate the development of such an application. A community of robotics researchers who use Blender for some simulations already exists, and there is a drive to improve on this functionality. Blender has BlenSor [72], a Free Open Source Simulation Package for Light Detection and Ranging (LiDAR/LADAR) and Kinect sensors

Cry Engine [73]: Since version 5.2, CryPhysics has supported multiple physical entity grids

In the case of simulation for a perception system, the most important component is the physics engine, which will allow modeling of the perception system of an autonomous vehicle with less fidelity. Physical simulators work according to the detection of collisions. These differ in the way they react in a collision. They usually work in two ways, where the collision is detected a posteriori or a priori. Collision detection refers to the problem of calculating the detection of the intersection of two or more objects.

As possible physics engines, we found Open Dynamics Engine (ODE) [74], a high-performance, open source library for dynamic simulation of rigid bodies. It is fully equipped, stable and with an easy to use C/C + + platform. It has some very useful methods, such as the method of approaching friction. An advantage is that it is a free and open source. ODE uses a Euler integrator and fixed time stepping. It provides an additional 2D constraint, and has been ported to a large number of platforms.

Bullet physics [75] is a powerful open source physics engine. It differs from other physics engines such as Box2D, Chipmunk, or Sprite Kit’s physics engine. This physics engine is 3D and includes 3D collision detection, soft body dynamics, and rigid body dynamics. It also includes a partial graphics processing unit (GPU) for physics implementation.

A very powerful and free physics engine is NVidia PhysX [76]. PhysX is a proprietary middleware or middleware layer engine and a development kit designed to perform very complex physical calculations. Physical middleware engines allow videogame developers to use abstraction during development, as PhysX provides specialized functions in complex physical simulations, which results in high code writing productivity.

### 5.3. Robotics Simulators

Robotics simulators are also used in the simulation of autonomous vehicles. Different works [77,78,79] mention that to be able to consider a robotics simulator useful in the domain of autonomous vehicles, they must provide modeling of all sensors and actuators present in an autonomous vehicle. They must also provide an environment as realistic as possible to simulate and test algorithms of acquisition and fusion of data from the sensors, navigational planning, and control of the steering and traction system. Bearing this in mind, the following criteria represent the most important aspects to consider when selecting a robotics platform in an autonomous vehicle approach:

- 3D rendering: The visual robustness of the simulation
- License: Whether the simulator has a General Public License (GPL), or a commercial one
- External Agent Support: In order to control a vehicle using an agent-based methodology, the simulator should feature a distributed architecture at the control level
- Sensor noise: Simulators that are able to calculate random noise at the outputs of the sensors will allow for more realistic testing of the decision-making systems that need to deal with non-ideal nature that real sensors have
- Parallelism/Distribution: In order to distribute processing power over processor cores or networks
- Level of Maturity: If the simulator is already widely used and validated
- Fault-tolerance: When a hardware module fails, higher level modules should rapidly make decisions whether to stop or modify the control system. When developing a final product, such behavior should be strictly tested
- Realistic Scenario Simulation: The level of realism to simulate difficult context scenarios e.g., snow, day and night, and wind, not only interactively but also physically, i.e., affecting the sensorial input:
  ○ Environment affecting sensors—harsh weather conditions and hazardous terrains can affect sensors in various ways; for example, fog or darkness affecting the visibility of an optical camera, or intense weather causing echoes in laser scanners. Simulators might include these factors in the calculation of sensor values
  ○ Environment affecting physics—weather and ground conditions can also affect the performance and control of the vehicle in various ways; for example, loose gravel, rain, or snow making the roads more slippery
- Techniques for HIL simulation have been recently applied to the automatic generation of complex controllers for robots. A robot uses its own real hardware to extract sensor and actuation data, then uses this data to infer a physical simulation (self-model) containing aspects such as its own morphology as well as characteristics of the environment. Algorithms such as Back-to-Reality (BTR) and Estimation Exploration (EEA) have been proposed in this context.

One of the key aspects to take into account when selecting a robotic simulation framework is the type of compatible sensors and implementation of their mathematical model. Some current robotics simulation platforms incorporate data simulation sensors such as Gazebo [80], V-REP [81], or Webots [82] (see Table 4 for a broader list). Another aspect to take into account is the widespread use of some frameworks or middleware in robotics (for example, ROS [83] or YARP), which make use of simulators conditioned by the characteristic of being compatible with the middleware. The Robot Operating System (ROS) is a mature and flexible framework for robotics programming. ROS provides the required tools to easily access sensors data, process that data, and generate an appropriate response for the motors and other actuators of the robot. The whole ROS system has been designed to be fully distributed in terms of computation, so different computers can take part in the control processes, and act together as a single entity (the robot).

Gazebo [80] is an open source simulator that offers the ability to simulate robot systems in complex environments. It is one the most popular simulation platforms for the robotic simulation research work. Gazebo has a modular design that allows different physics engines to be used, along with high-quality graphics, sensor models, and the creation of 3D worlds and graphical interfaces. Gazebo is built on top of the rendering engine Ogre3D to provide more realistic environments. The use of plugins expands the capabilities of Gazebo to include abilities such as dynamic loading of custom models and the use of stereo cameras, LiDAR, GPS, IMU or RADAR sensors. Being an open source product, there is a large and active robot community supporting and improving the product. One of the advantages of Gazebo is that it is already included in the ROS bundle. Gazebo is informally part of the ROS, a set of libraries and open source software tools that allow the user to create a robotic application. ROS and Gazebo are becoming more popular for the development of AVs. There have been a multitude of studies designing methods for validating the physics and sensor simulations of Gazebo; for example, in the latest edition of ROSCon Conference [84], BMW presented several works [85,86] that focus on the simulation of autonomous vehicles. The TSC prototype Basic Autonomous Control Systems (B-ACS) for the LUTZ pods has been developed using ROS and simulated using Gazebo.

The robot simulator V-REP [81], with an integrated development environment, is based on a distributed control architecture. Each object/model can be individually controlled via an embedded script, a plugin, a ROS or BlueZero node, a remote API client, or a custom solution. This makes V-REP very versatile and ideal for multi-robot applications. Controllers can be written in C/C++, Python, Java, Lua, Matlab or Octave.

Webots is a commercial robot simulator developed by Cyberbotics used in more than 800 universities and research centers worldwide. It has reached a fairly stable state and supports a wide range of hardware. Webots makes use of ODE (Open Dynamics Engine) for the detection of collisions and dynamic simulation of the rigid body. The ODE library allows the physics of the objects to be simulated. Note that the physics plugins can be programmed only in C or C++. The software also provides a large collection of sensors, including a distance sensor, light sensor, cameras, LiDARs, GPS, accelerometer, and force-sensor.

Microsoft Robotics Developer Studio (MRDS) is a Windows-based robotics platform from Microsoft Company using .NET based technology. It features visual programming, web and windows-based interfaces, 3D simulation with advanced physics, as well as easy access to robot’s sensors and actuators in a number of languages. In addition to providing support for Microsoft Visual Studio 2010, Microsoft Robotics Developer Studio 4 provides a Visual Programming Language (VPL), which allows developers to create applications simply by dragging and dropping components onto a canvas and wiring them together. Princeton University’s DARPA Urban Grand Challenge Autonomous Car [87] was programmed with MRDS. It is oriented towards educational projects with a low degree of complexity.

Apart from robotic simulation platforms, there are other simulators in the robotic domain, which are relevant for research. Among them are USARSim [88], BlenSor [72] and MORSE [89]. USARSim is a free simulator based on the cross platform Unreal Engine. It was released under the GPL license, physics is simulated using the Karma Physics. USARSim comes with several detailed models of robots available for use in simulations; however, it is possible to create custom robot components in external 3D modeling software and specify physical attributes of the components once they are loaded into the simulator. BlenSor is a Free Open Source Simulation Package for Light Detection and Ranging (LiDAR/LADAR) and Kinect sensors. BlenSor is not designed for real-time simulation, as it is a precise simulation and with complex scenarios, it takes time. BlenSor is not based on the Blender game engine (or any other) and can simulate complex scenarios. It is designed to produce data for offline data processing. They are designed for the simulation of robotic environments and also implement a laser range scanner. MORSE is a generic simulator for academic robotics. In MORSE, simulations are small Python scripts that describe the robots and the environment. MORSE provides several command-line tools to create stubs, and it takes virtually no time to get a first simulation running. It provides a set of standard sensors (cameras, laser scanner, GPS, odometry, ...), actuators (speed controllers, high-level waypoints controllers, generic joint controllers) and robotic bases (quadrotors, ATRV, Pioneer3DX, generic 4-wheel vehicle, PR2, ...). MORSE is designed to interact directly with the evaluated software exactly as it is, without the need of any modifications to the software. This philosophy takes after the “Hardware-in-the-Loop” simulations, in which the evaluated components are run on the target hardware and interact with the simulator with the very same protocols than the ones of the actual robot sensors and actuators, in order to make the shift from simulations to actual experiments totally transparent. In Table 4, we compare the main features of these robotic simulator platforms.

In Table 5, we can compare which sensors are simulated by these generic robot simulators and the sensors equipped.

### 5.4. Perception Simulator 

As can be seen in the previous sections, there are multiple generic solutions to validate and simulate an autonomous vehicle (using simulators in the loop, using a robotics platform, or a game engine among others). Because an autonomous vehicle bases all its decision-making on the feedback received from the sensors, it is clear that the virtualization of the different sensors is key to any simulation platform for autonomous vehicles. However, for a simulation to be the most identical to reality, perfect models are needed to minimize the reality gap [90,91]. However, in some cases, the results obtained with generic simulators do not reach the expected degree of satisfaction or simply do not contemplate all the causality that is needed. For these cases, the best option is to develop a virtual simulation environment specifically dedicated to one’s own needs.

**Figure 8:**
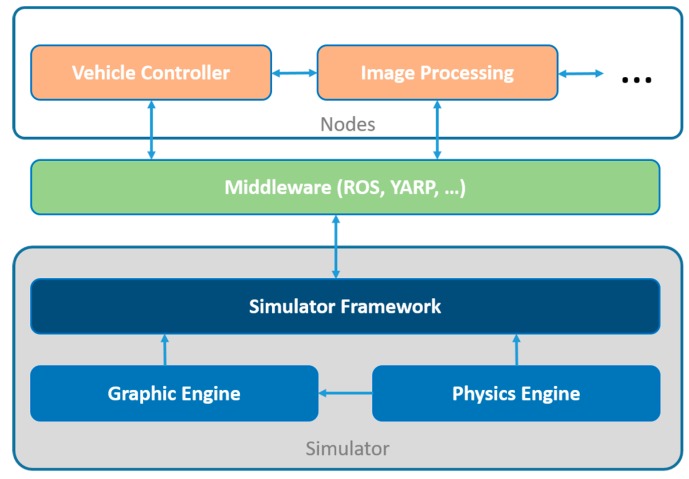
Typical software architecture of a simulator.

The virtual environment consists of an approximate vehicle model or sensor model, a simulated world and simulated sensors. The most important component that creates a virtual environment is the simulator. In Figure 8, the typical software architecture of a simulator is shown.

In some cases, a simulator that realistically represents the physics of an environment — things like force and mass, friction and air drag — is sufficient. For development of perception algorithms, however, a simulator must feature a photorealistic representation of the environment, meaning that the simulated environment must actually look like the real world.

Modern simulators tend to provide the following features:

- Fast prototyping
- Physics engines for realistic movements. Most simulators use Unity (AirSim), Unreal Engine (Carla, USARSim), ODE (Gazebo, LpzRobots, Marilou, Webots) or PhysX (MRDS, 4DV-Sim).
- Realistic 3d rendering. Standard 3d modeling tools or third party tools can be used to build the environments.
- Dynamic with scripting. C, C++, Perl, Python, Java, URBI, MATLAB or Python are a few of the languages usually used with the most common simulators.

In Table 6 and Table 7, we compare the main features of these simulators and which sensors can be simulated by these simulators and the sensors equipped. Many of the simulators oriented towards autonomous vehicles found in existing literature are intended for traffic simulators and driving simulators [85,92,93,94,95,96,97] that allow training and validating autonomous driving from an algorithm and artificial intelligence point of view. The Agent paradigm has been introduced as well, as a way to address some of the current issues in autonomous-based driver behavior regarding its distribution capabilities, computing efficiency and scalability Agent-based modelling and simulation (ABMS) is a relatively new approach to modelling systems composed of autonomous, interacting agents. Agent-based modelling is a way to model the dynamics of complex systems and complex adaptive systems. Such systems often self-organize themselves and create emergent orders. Agent-based models also include behavior models (human or otherwise) and are used to observe the collective effects of agent behaviors and interactions. The development of agent modelling tools, the availability of micro-data, and advances in computation have made a growing number of agent-based applications possible across a variety of domains and disciplines. These simulators can be used in the design and simulation of ADAS and that is why some of these simulators also include a perception subsystem, although it is usually a secondary part of the simulator, with models of pseudo-sensors or in the best of cases with a basic LiDAR model (given the importance of this sensor in autonomous vehicles). Although these driving simulators are not the objective of this work, the most significant ones have been included as an example.

It should be noted that main vehicle manufacturers are working on their own prototypes of autonomous vehicles and also have their own products for validation, verification, testing and simulation. 

Toyota has its own vehicle Platform3.0, developed by the Toyota Research Institute (TRI); the platform is built on a Lexus LS 600. The main advancement of the Platform3.0 is in its sensor systems, which have increased in quantity and definition, converting this model in an automated car to the greatest perception capacity. It uses a LiDAR system with 360 degrees of vision and a range of 200 m. Toyota has also collaborated in the development of the CARLA simulator [93]. BMW is another brand that is betting strongly on autonomous technology. BMW iNext is the brand’s proposal. For simulation, they have opted for a robotics framework and middleware. BMW has even established its own Autonomous Driving Campus in Munich [106]. 

FORD and SEAT are also in the list of vehicle brands that are committed to autonomous driving.

On the other hand, companies in the electronics, computer and information technology sectors such as Apple or Google also have their own proposals. Google has developed Wymo with level 4 autonomy. Google has a series of proprietary tools, among which we highlight the Carcraft simulator [101], which supports a large number of sensors (RADAR, LiDAR, ultrasonic, ...). NVIDIA, one of the most important GPU development companies (Graphics Processing Unit) has its own autonomous vehicle development platform (NVIDIA DRIVE AGX), NVIDIA DRIVE [107], which offers specialized software for sensor perception and fusion, and complements the NVIDIA DRIVE CONSTELLATION ™ simulator. NVIDIA DRIVE Constellation ™ is a data center solution that integrates powerful GPUs and DRIVE AGX Pegasus ™. The advanced visualization software running on GPUs simulates cameras, radars and LIDARs as inputs to DRIVE AGX Pegasus, which processes the data as if you were actually driving on the road.

The efforts required to bring autonomous cars to the masses are so immense that car and technology companies are unable to achieve it alone. The Volkswagen Group of America and major players in the automotive innovation industry like Bosch and NVIDIA have joined forces to pool their resources and expertise in a bid to accelerate the development of driver-less technology.

## 6. Legislation 

### 6.1. Standardization Concepts for Autonomous Driving

The systems that provide the autonomous driving capabilities are formed by sensors and actuators that communicate through networks and are controlled by microcontrollers, which makes software a key factor. With tens of millions of lines of code (code for applications, operating systems and middleware), aspects related to security and protection arouse great concern. In order to regulate the aforementioned aspects, the specific standard for car protection ISO 26262 [108] has been developed. It is an adaptation of the functional protection standard IEC 61508, which focuses on the needs of the electrical and electronic systems installed in passenger cars, and applies to all activities within the lifecycle of these systems related to protection, including software quality requirements.

The standard uses Automotive Safety Integrity Levels (ASIL) to offer a measure of the risk associated with a subsystem. These levels go from A to D, A being the lowest level of integrity and D the highest, that is, the most demanding with the most requirements.

The parameters of risk severity, probability of exposure, and controllability determine the ASIL. The parameter of controllability requires special attention. It is assumed that the driver is in proper driving conditions, has the proper training for driving (driving license) and complies with all applicable legal regulations, including corresponding requirements to avoid risks with other road users; the driver must comply with traffic laws. It is necessary to adapt the laws, so that when an automatic driving system is in operation, the driver does not have to pay attention, unless the system requests his intervention. The correct operation of the notification to the driver and the request for human control is essential. If the notification fails, it is possible that the human driver is not paying attention and cannot avoid the danger. If the request fails, the system should continue to carry out the control instead of allowing the driver to intervene.

These situations should always be assigned to the highest control class (C3), which means that less than 90% of drivers or other road users are generally able, or barely able, to avoid the hazard. Part 6 of the 26262 standard is dedicated to the software development process to produce code reliable enough to run a system and meet the required ASIL level. 

The J3016 standard of the SAE (Society of Automotive Engineers) divides the driving automation into six classes: from non-automatic (level 0) to fully automatic (level 5). SAE levels 3 or higher, are based on software to collect data from the sensors in order to create a model of the environment and then, depending on the objective, decide on how to assist the driver or control the vehicle. It also covers other critical tasks, such as determining if the sensors are working correctly, when to notify the driver and when to activate the request for human control. It is vital that this software responds reliably. Other software tasks, such as modeling the sensor data, may be less critical, but even this will require analyzing the risk.

The aforementioned safety and protection aspects for all the agents involved in the automation of driving are being analyzed in depth by the competent authorities in traffic and road safety. In the following section, an analysis of the current legislation regarding autonomous driving is made.

### 6.2. Legal Aspects of Autonomous Driving

Like all the technologies that allow the automation of tasks performed by humans, autonomous driving is perceived by them as a generator of different risks, which can hinder its acceptance; we can classify these risks as technological risks, social risks, economic risks and adaptation risks [109]. Among potential technology risks, Renn [110] highlights five: Security, liability, privacy, cybersecurity and industry influence. The responsibility of minimizing the impact of the aforementioned risks falls on the different governments, the only ones with the capacity to generate the appropriate policies and legislation; the legislators work on it at different rates, as we explain below.

The legislation that is being produced to regulate the use of autonomous driving focuses mainly on levels 4–5 of AV automation as proposed by the Society of Automotive Engineers (SAE) and tries to overcome the biggest legal obstacle established by the United Nations Vienna Convention on road traffic, which forces the control of the vehicle to be in the drivers’ hands permanently and under any circumstance.

Table 8 shows a list of countries whose governments are decisively involved in generating legislation to regulate the circulation of autonomous vehicles, the state of legislation (approved, billed or drafted) and legislative orientation, according to Li’s proposal [109].

### 6.3. AV Testing Possibilities

As explained in the previous section, the various factors involved in the regulation of AV circulation means the development of legislation that regulates this activity becomes a complex and slow task. This difficulty largely hampers the deployment of a technology that has already reached a high degree of maturity.

One of the main issues presented by the lack of legislation is limitation in the testing of autonomous vehicles on roads open to traffic.

As summarized in Table 9, only a small set of 17 countries/states around the world allow partial access to public roads by autonomous vehicles for testing purposes and, among them, only 11 allow unrestricted access.

Legislation limiting liability for possible accidents caused by autonomous vehicles is also concerned with establishing standards for the development of tests in real traffic conditions. Thus, institutions such as the European Road Transport Research Advisor Council (ERTRAC, Europe), the National Highway Traffic Safety Commission (NHTSA, USA), the Center for Connected and Autonomous Vehicles (CCV, UK) or the National Transport Commission (NTC, Australia) establish conditions under which the tests must be carried out. In general, the established rules are very restrictive in that it is necessary to communicate in advance the nature of the tests (details of the tests to be performed, equipment, routes, safety measures adopted, etc.).

All this generates great complexity, both in the administrative procedures and necessary security deployment, which causes the tests to be expensive and not as abundant as would be desirable by the developers of this technology. That is why companies make great use of simulation techniques for the development of systems for autonomous driving.

## 7. Conclusions

Advances in the field of vehicles with a high degree of automation are growing very rapidly, as can be seen from the study carried out in this review on perception systems. Every so often, new sensors appear (e.g., solid state LiDAR or MMW RADAR,) applied to autonomous driving or technologies that were unthinkable a few years ago beyond the use of research centres or large companies (e.g., rotational 3D LiDAR). The different electromagnetic spectrum bands used by the sensors studied in this work give each type of sensor advantages and disadvantages that limit its application. For example, MMR is used for tracking objects, calculation of relative speeds or in ADAS systems for detection of objects in blind spots; they also have an excellent response in all types of weather conditions. 3D LiDARs have a high spatial resolution and high precision that make them the perfect element for navigation and mapping the environment. Visible cameras have a chromatic reproduction that makes them vital when it comes to discerning between the objects present on the road (pedestrians, vehicles, signs, etc.). The new generation of thermal cameras allow operation in total darkness or in very bright environments with total precision. The analysis of the spider charts shown in Figure 7 shows that there is no perfect sensor, so it is necessary to carry out sensorial fusion by means of specific algorithms that make it possible to alleviate the defects of each sensor and combine their advantages. We must highlight the good results that are being obtained in the area of data fusion with CNN. In the area of positioning systems, RTK systems that offer centimetre accuracy at the expense of using base stations that correct the GNSS signal should be highlighted. In this area, there are efforts in the deployment of this type of infrastructure to give precise coverage to the new fleet of autonomous vehicles that in a short space of time will flood cities.

Autonomous driving is a challenge that involves different risks for road users. The deployment of technology that enables the automation of driving requires very exhaustive tests in real driving and road conditions, tests that in most countries cannot be carried out either in the quantity or in the actual driving conditions that would be required due to the absence of appropriate legislation, as explained in Section 6. This gap is largely covered by simulation.

As observed in Section 5, the simulation possibilities of an autonomous vehicle are very broad. Over 200 works have been found related to the simulation of autonomous vehicles. The options are reduced when the simulation is focused on the Perception System, in this case around 50 of the works consulted. Not all the works have been studied, only those with an impact factor (publications in congresses or research journals, which have passed a peer review) have been taken into account. On the other hand, we consulted the proposals offered by companies dedicated to modelling and simulation, robotics, and automation.

Undoubtedly, the most widespread simulators used in the field of research are robotics simulators. This is logical, considering that autonomous vehicles are a branch of robotics. However, not all robotic simulators are prepared to provide the necessary realism required in these cases. That is why the tendency is to use customized solutions using existing modelling and simulation platforms.

## Figures and Tables

**Table 1:** Summary of the main features of sensors used in perception systems of AV.

	Ultrasonic	RADAR	3D LiDAR		Cameras		
			Rotating	Solid State	VIS	IR	ToF
FOV	1	2	3	2	3	3	2
Range	1	3	3	3	2	3	2
Accuracy	1	2	3	3	3	2	2
Frame rate	2	2	2	2	2	3	3
Resolution	1	1	2	2	3	1	1
Colour perception	0	0	1	2	3	1	1
Size	1	1	2	1	1	1	1
Weather affections	1	1	2	2	3	1	3
Maintenance	2	1	2	1	2	2	2
Visibility	2	1	3	2	2	2	2
Price	1	2	3	1	1	3	2

**Table 2:** Most commonly used global positioning systems.

	GPS	GLONASS	GALILEO	BEIDOU
Satellites	24	24	30	30 + 5*
Precision	7.8 m, civil 5.9 m, military	7.4 m, civil 4.5 m, military	1.0 m, civil 0.01 m, advantage	10 m, civil 0.1 m, military
Coverage	Global	Global	Global	Chinese
Period	11 h 58 m	11 h 15m	14 h	12h 53m
height	26,650 Km	19,100 Km	23,222 Km	21,150 Km
Owner	EEUU	Russia	European Union	China

* Geostationary Satellite.

**Table 3:** Summary of the main features of simulator platforms for AV.

Simulators				XIL		
	License	Open Models	ISO 26262 Compliant	MIL	SIL	HIL
PaTAVTT [63]	GPL	x	U			x
Simulink &Matlab [61]	Commercial	-	X	x	x	x
dSpace GmbH [56]	Commercial	-	X	x	x	x
LabVIEW [64]	Commercial	-	X	x	x	x
CarSim [65]	Commercial	u	X	x	x	x
CAT Vehicle [66]	GPL/Open Source	x	U	x	x	x

* Table Legend: x - Yes | u – Unknown or couldn’t be determined | - – No.

**Table 4:** Summary of the main features of robotic simulator platforms for AVs.

Simulator Platforms	License	Simulation Engine	Graphical Engine	External Agent
Gazebo	GPL/Open Source	ODE, Bullet, Simbody Art	Ogre3D	Yes
V-Rep	GPL/Open Source, Commercial	ODE, Bullet, Vortex	OpenGL	Yes
Webots	Commercial	ODE	-	Yes
MRDS	Commercial	PhysX	DirectX	No
USARSim	GPL	Unreal Engine	Karma	Yes
BlenSor	GPL/Open Source	-	OpenGL	No
MORSE	GPL/Open Source	Blender, Bullet	OpenGL	Yes

**Table 5:** Summary of the main sensors simulated by robotic simulator platforms for AVs.

Simulator Platforms	GPS	IMU	LIDAR	Ultrasonic	Radar	Infrared	Stereo Camera	ToF Camera
Gazebo	x	x	x	x	x	x	x	x
V-Rep	x	x	x	x	x	x	x	u
Webots	x	u	x	x	-	x	x	-
MRDS	x	u	x	x	u	x	u	u
USARSim	x	x	x	x	x	x	x	u
BlenSor	x	x	x	x	u	u	x	x
MORSE	x	x	x	-	-	x	x	u

* Table Legend: x - Yes | u – Unknown or couldn’t be determined | - – No.

**Table 6:** Summary of the features of specific simulators for AVs.

Simulator	License	Physics Engine	Graphic Engine	Scripting Language	External Agent	Notes
CARLA [93]	GPL/ Open Source	Unreal Engine	GPU	Python	Yes	Driving
AirSim	GPL/ Open Source	Unreal Engine	u	C++, Python, C#, Java	Yes	Driving/HIL,SIL
DeepDrive [98]	GPL/ Open Source	Unreal Engine	u	C++, Python	Yes	Driving
Udacity * [99]	GPL/ Open Source	Unity	u	C++, Python	u	Driving
Constellation [100]	Restricted	PhysX/CUDA	GPU	C/C++, Python	Yes	Cloud, HIL, VR
Carcraf/Waymo [101]	Restricted	u	u	u	Yes	Driving
SIMLidar [102]	GPL/ Open Source	u	u	C++	u	LiDAR
Helios [103]	GPL/ Open Source	JMonkey Engine	OpenGL	Java	u	LiDAR
GLIDAR [104]	GPL/Open Source		OpenGL	C++	u	LiDAR
RADSim [105]	Comercial	u	u	MATLAB	u	RADAR
SIMSonic	GPL/Open Source	u	u	R	u	Ultrasonic

* Table Legend: u–Unknown or could not be determined.

**Table 7:** Summary of the main sensors simulated by specific simulators for AVs.

Simulator	GPS	IMU	LIDAR	Ultrasonic	Radar	Infrared	Stereo Camera	ToF Camera
CARLA	x	-	x	-	-	-	x	-
AirSim	x	x	x	u	U	u	u	u
DeepDrive	x		x	-	X			-
Udacity *	x	x	x	u	U	x	u	u
Constellation	x	x	x	x	X	x	x	u
Carcraft/Waymo	x	x	x	x	X	x	x	u
SIMLidar	-	-	x	-	-	-	-	-
Helios	-	-	x	-	-	-	-	-
GLIDAR	-	-	x	-	-	-	-	-
RADSim	-	-	-	-	X	-	-	-
SIMSonic	-	-	-	x	-	-	-	-

* Table Legend: x-Yes | u–Unknown or could not be determined | - – No.

**Table 8:** Summary of the main AV legal regulation.

Country	Prevention Oriented	Control Oriented	Toleration Oriented	Adaptation Oriented
Australia (NTC)	Approved Bill Draft	Bill, 2017 Approved, 2016 Onboard driver		Draft, 2017
China (NTCAS)		Bill, 2016 Approved, 2016		
France				
Germany		Approved, 2017	Draft, 2017	
Japan	Approved, 2016 Onboard driver			
New Zealand				
South Korea		Approved, 2016		
Sweden				
The Netherlands		Approved, 2016		
Singapoore (RTA)		Approved,2017 No driver		Bill, 2017
UK (CCAV)			Approved, 2017 Onboard driver	Draft, 2018
USA (Alaska) (NHTSC)		Approved, 2016		
USA (Arizona) (NHTSC)		Approved, 2016		
USA (California) (NHTSC)		Approved, 2015		
USA (Florida) (NHTSC)		Approved, 2015		
USA (Nevada) (NHTSC)		Approved, 2017		
USA (rest of States)		Bill, 2018		

**Table 9:** Permitted access to public roads for AVs.

Country	No Access	Partial Access	High Access
Australia		X	
China		X	
France		X	
Germany			X
Japan		X	
New Zealand			X
South Korea			X
Sweden			X
The Netherlands			X
Singapore			X
UK		X	
USA (Alaska)			X
USA (Arizona)			X
USA (California)			X
USA (Florida)			X
USA (Nevada)			X
USA (rest of States)		X	
Remaining countries	X		
